# Markers of lutein and zeaxanthin status in two age groups of men and women: dietary intake, serum concentrations, lipid profile and macular pigment optical density

**DOI:** 10.1186/1475-2891-13-52

**Published:** 2014-06-03

**Authors:** Begoña Olmedilla-Alonso, Beatriz Beltrán-de-Miguel, Rocío Estévez-Santiago, Carmen Cuadrado-Vives

**Affiliations:** 1 Department of Metabolism and Nutrition, Institute of Food Science, Technology and Nutrition (ICTAN-CSIC), C/José Antonio Novais, 10, 28040 Madrid, Spain; 2 Department of Nutrition, Faculty of Pharmacy, Complutense University of Madrid, 28040 Madrid, Spain

**Keywords:** Lutein, Zeaxanthin, Serum, Dietary intake, Macular pigment optical density, Lipid profile

## Abstract

**Background & aims:**

Lutein and zeaxanthin accumulate in retina (macular pigment). Their nutritional status can be assessed using dietary or biochemical markers and both have been associated with macular pigment optical density. We proposed to assess dietary and status markers of lutein and zeaxanthin in a group of healthy Spanish volunteers, considering the potential influence of age, gender and serum lipids to investigate the predictors of the macular pigment optical density.

**Methods:**

Serum lutein and zeaxanthin concentrations, dietary intake and macular pigment optical density were determined in 108 healthy men and women (20–35 and 45–65 years), using high-performance liquid chromatography, 3-day food records and heterochromic flicker photometry, respectively. Mann–Whitney U-test, Spearman correlation coefficient and multivariate regression analysis were used for the statistical study.

**Results:**

Serum concentrations and dietary intake of lutein plus zeaxanthin (p < 0.0001 and p = 0.001, respectively) were higher in older vs younger subjects, whereas macular pigment optical density was lower (p = 0.038). The highest correlation coefficients between intake and serum were for fruit and serum lutein (ρ = 0.452, p < 0.0001) and for fruit and lutein + zeaxanthin (ρ = 0.431, p < 0.0001) in the younger group. Macular pigment optical density correlated with serum xanthophylls (ρ = 0.223, p = 0.02) and fruit and vegetable intake (ρ = 0.350, p = 0.0002), showing highest correlations when lutein and zeaxanthin were expressed in relation to serum lipids in older subjects (ρ = 0.262, p = 0.006). Multivariate regression analysis identified age and serum lutein as major predictors of macular pigment optical density (total sample), and a coefficient of determination of 29.7% for the model including lutein + zeaxathin/cholesterol + triglycerides, sex and fruit + vegetables in the older group.

**Conclusions:**

The establishment of normal/reference ranges for serum lutein and zeaxanthin should consider age ranges and be expressed in relation to lipid concentrations, at least in subjects over 45 years, as this could influence macular pigment optical density. The macular pigment optical density showed age-specific correlations with lutein plus zeaxanthin expressed in relation to serum lipid concentrations as well as with the fruit and vegetable intake.

## Introduction

Lutein and zeaxanthin are plant pigments that belong to the well-known group of carotenoids, subgroup of xanthophylls (oxycarotenoids), found in the human body (blood, tissues, and concentrated in retina, where they constitute what it is known as the macular pigment [MP]). They are provided only in the diet, and are transported in blood to the different tissues by lipoproteins [1]. Both xanthophylls, but primarily lutein, have been investigated in relation to eye health and disease. They act by filtering blue-light and as antioxidants that may protect macular pigment from oxidative damage induced by light and the high rate of oxidative metabolism in the eye. Moreover, there is increasing evidence suggesting that MP may protect against age-related maculopathy [2].

Traditionally, exposure to or nutritional status assessment of lutein (generally assessed together with zeaxanthin) in human subjects has routinely been performed using dietary or biochemical methods [2-5]. The dietary intake of lutein and its presence in blood / tissues (i.e. in retina) have been associated with a lower risk for age-related diseases, and the levels increase upon the ingestion of lutein-rich foods and lutein supplements [3,6,7]. Determination of serum lutein and zeaxanthin concentrations has been considered the best available method for establishing their nutritional status in human subjects [2,4,5], and these concentrations are affected by dietary and host-related factors that influence their absorption and utilization [3,7]. The lutein concentration in serum is considered to reflect short-term dietary intake and, although it often correlates poorly with said intake, it is widely accepted as a good biomarker of fruit and vegetable intake [3].

The MP density can be considered a marker of long-term dietary exposure [4,8] that can be measured by a number of techniques and the most widely used noninvasive test is heterochromatic flicker photometry [9]. Its assessment is increasingly being performed due to new commercially available devices that can easily be employed in clinical practice. To know to what extent macular pigment optical density (MPOD) can be considered a surrogate measure of lutein and zeaxanthin intake and status, we need more data from well-defined, homogeneous populations, with information on the many factors affecting the levels of these compounds. This would shed light on their role in visual function and in the reduction of risk of eye diseases, and allow comparisons between population groups. Dietary and serum lutein and zeaxanthin concentrations have often been positively associated with MP [5,7,8], but conflicting results have been reported concerning the influence of several variables (sex [2,9], age [2], body mass index (BMI) [10] and lipids [11]), among other factors.

This study was designed to assess: 1) dietary and status markers (diet, serum and MPOD) of lutein and zeaxanthin, together with serum lipid profile as a confounding factor and, 2) the predictive value of those compounds and several confounding factors (age, sex, serum lipids) for the MPOD, in a population of well-characterized healthy Spanish volunteers, grouped according to age and gender, as these factors can modify the lutein and zeaxanthin concentrations in the three status markers or the correlations among them.

## Materials and methods

### Subjects and study design

108 volunteers (54 men and 54 women), divided into two age groups (20 to 35 y and 45 to 65 y) (mean ± SD: 25.6 ± 3.2 y and 52.4 ± 5.2 y, respectively) were enrolled in a cross-sectional study. These age groups were established because of their different dietary habits and risk of age-related ocular disease. Participants were selected from those subjects who were interested and contacted through advertisements in different universities, research centers, and several noticeboards (i.e. intranet of several Spanish ministries). Of the 137 individuals who showed their interest in participating in the study, 5 were excluded because of a high cholesterol level, 4 because they were taking omega-3 food supplements or enriched milk, 2 because of his/her BMI and 19 because their age and sex corresponded to a group in which the established number of subjects had been reached. The inclusion criteria were normal cholesterolemia, BMI under 30 kg/m^2^, mixed diet (no avoidance of any food groups). Volunteers were asked to report information on the following exclusion criteria: consumption of dietary supplements, BMI under 20 kg/m^2^, surgery for myopia (within the previous year), cataracts or macular degeneration, use of drugs or phytosterol-enriched beverages/foods to control cholesterol level, regular consumption of n-3 fatty acid-enriched food products and chronic diseases that can affect carotenoid or lipid metabolism (i.e. diabetes, cardiovascular disease).

The volunteers included in this cross-sectional study underwent blood sampling, assessment of the MPOD and 3-day food records. The subjects were enrolled over the course of an entire year (during the spring and summer: 40 in the younger and 29 in the older age group, and, during the fall and winter: 14 in the younger and 25 in the older age group). Blood samples were collected after overnight fast (at least 8 hours) and serum obtained for the analysis of lutein, zeaxanthin and lipid profile.

This study was conducted in accordance with the guidelines laid down in the Declaration of Helsinki and all procedures involving human subjects were approved by the Clinical Research Ethics Committee of Hospital Universitario Puerta de Hierro-Majadahonda of Madrid, Spain (registry no. 257, dated 19 July 2010). Written informed consent was obtained from all subjects.

### Lutein, zeaxanthin and lipid analysis in blood

Lutein and zeaxanthin levels were determined by high performance liquid chromatography (HPLC) using a system consisting of a model 600 pump, a Rheodyne injector and a 2998 photodiode array (PDA) detector (Waters, Milford, MA, USA) in accordance with standard procedures described elsewhere [12]. We used a Spheri-5 ODS 5 μm (220 mm × 4.6 mm) chromatographic column (Brownlee Labs, Applied Biosystem, Santa Clara, CA, USA) with a guard column (Aquapore ODS type RP-18). The mobile phase was acetonitrile-methanol (85:15; v/v), and was changed to acetonitrile-dichloromethane-methanol (70:20:10; v/v/v) in a linear gradient from min 5 to min 20. Both mobile phases were stabilized with ammonium acetate (0.025 mol L^−1^) added to the methanol. The flow rate was 1.8 mL min^−1^, and detection was performed at a wavelength of 450 nm. All chromatograms were processed using Empower 2 software (Waters, Milford, MA, USA).

Carotenoid extraction was performed on serum samples using a slight modification of a previously published method [12]. Briefly, 200 μl of serum was added to 200 μl of ethanol, vortexed and extracted twice with 400 μl of hexane: dichloromethane (5:1) stabilized with 0.1 g/L butylated hydroxyltoluene. Organic phases were pooled, evaporated to dryness under nitrogen atmosphere and reconstituted with 200 μl of a solution of tetrahydrofuran: ethanol (1:2) and injected (5 μl) onto the HPLC system.

Methanol, ethanol, acetonitrile, dichloromethane, ammonium acetate, butylated hydroxytoluene (BHT) and tetrahydrofuran were supplied by Panreac (Barcelona, Spain). Lutein (xanthophyll from marigold) was obtained from Sigma Chemical Co. (St. Louis, MO, USA) and zeaxanthin was purchased from Fluka Analytica (Sigma Aldrich).

Standard solutions were prepared from 1 mg of lutein and of zeaxanthin dissolved in 25 mL tetrahydrofuran, with 0.01% BHT in each case. The *E*_1cm_^1%^ values and wavelengths used were as follows: lutein, 2550 at 445 nm; zeaxanthin, 2540 at 450 nm. Working solutions were obtained from different volumes of the standard solutions dissolved in tetrahydrofuran: ethanol (1:2 v/v). The concentrations of the carotenoids in the curve were: 0.27-1.36 μg mL^−1^ for lutein (*R*^2^ = 0.999) and 0.03-0.15 μg mL^−1^ for zeaxanthin (*R*^2^ = 0.999).

Blood total cholesterol and high-density lipoprotein (HDL) cholesterol were analyzed by colorimetric enzyme assay (Cobas Integra 400 plus, Roche). The low-density lipoprotein (LDL) cholesterol level was calculated with the Friedewald *al* equation [13]. Serum triglycerides were determined using an enzymatic colorimetric test (Roche Diagnostics, GmbH Manheim, Germany), a method that employs lipase, glycerol kinase, glycerol-3-phosphate oxidase and peroxidase.

### Dietary intake assessment

Recent dietary intake was evaluated using 3-day food records involving 24 h recalls, one of which coincided with a weekend or holiday, carried out within a period of 7 to 10 days. For the first recall, the participants underwent a face-to-face encounter with a specialized interviewer, normally the same person who, subsequently, performed the other two recalls by telephone. The amounts consumed were estimated in units (fruits), portions or household servings standardized for this study [14]. On the basis of this information, we calculated food intake in grams/day, which served as the basis for the determination of the daily lutein and zeaxanthin intake using a database designed by our group, included in a software application for the calculation of dietary intake of individual carotenoids [15]. To evaluate the lipid and energy intake, we employed a food composition table widely used in Spain [14].

### Macular pigment optical density (MPOD) assessment

Macular pigment optical density was assessed using an MPS 9000 desktop device (Macular Pigment Screener, Elektron PLC, Cambridge, UK) that applies the principles of heterochromatic flicker photometry. The technique and reliability of this device are described in detail by van der Veen et al. [16]. The test consists of two stages for central and peripheral viewing, and the subjects were required to press a response button as soon as they detect flicker. The subjects started by fixating the central stimulus, a 1-degree central target (flicker rate is initially set to 60 Hz and then gradually reduced at a rate of 6 Hz s^−1^). The process was repeated for a series of green-blue luminance ratios. The observer then fixated a red 2°-diameter target placed 8° eccentrically and a second set of data were recorded for peripheral viewing [17]. The MPOD is measured in density units (du) and ranges from 0 to 1.

### Statistics

Sample size calculation was performed on the basis of a mean value for MPOD of 0.40 du. A sample of 108 subjects (SD = 0.10) was found to be necessary to obtain a 10% difference in the MPOD (0.04 du) with 85% power and an alpha error of 0.05. Data are expressed as the mean and standard deviation, median and 95% confidence interval. The normal distribution of the data was assessed (Kolmogorov-Smirnov test) and, as lutein and zeaxanthin in serum and diet and serum triglycerides did not follow a normal distribution, the Mann–Whitney U test was used to compare concentrations of the variables analyzed in the two groups (established according to sex and age). Age and sex were introduced as covariates in the generalized linear model and only age affected the concentration/intake of several variables. No interactions were observed for any of the variables except for serum lutein + zeaxanthin/HDL serum which was affected by age and by sex and, HDL-cholesterol which was influenced by sex.

Correlations among variables in serum, diet and the MPOD were established using Spearman’s rho correlation coefficient. All reported *P*-values are based on a two-sided test and compared to a significance level of 5%.

Multiple linear regression analysis was carried out using backward elimination as a model selection procedure, with macular pigment optical density as dependent variable and the following independent variables: sex, age, lutein and zeaxanthin in serum and in diet, lutein + zeaxanthin in serum and in diet, lutein + zeaxanthin/cholesterol + triglycerides, lutein + zeaxanthin/HDL-cholesterol, lutein + zeaxanthin/LDL-cholesterol and fruit and vegetable consumption. SPSS v.20 (SPSS Inc., Chicago, IL, USA) software was used for all statistical calculations.

## Results

Table 1 shows the concentration of lutein and zeaxanthin in diet and serum, serum lipids, energy intake, MPOD and BMI of the sample as a whole and grouped according to age and sex, expressed as the mean and standard deviation, median and confidence interval. While there were no differences in lutein and zeaxanthin in serum and diet when the sexes were compared, we did observe differences between the two age groups, with higher concentrations in the older subjects than in the younger subjects. The lutein concentration was higher than the zeaxanthin concentration in both dietary intake (10.8-fold) and serum (4.6-fold) in the total sample (median values).

**Table 1:** **Dietary intake and serum concentrations of lutein and zeaxanthin, macular pigment optical density, serum lipids and body mass index in Spanish subjects (n=108) expressed as mean ± standard deviation, (median) and confidence interval [**_**95%**_** CI]**

	**Men**	**Women**	**Aged 20–35 years**	**Aged 45–65 years**	**Total sample**
	**(n = 54)**	**(n = 54)**	**(n = 54)**	**(n = 54)**	**(n = 108)**
***Concentrations in serum***					
**Lutein** (μg/dl)	13.0 ± 6.9	12.7 ± 5.5	10.9 ± 5.0^b^	14.8 ± 6.6^b^	12.8 ± 6.2
	(11.2)	(11.7)	(10.0)	(13.0)	(11.6)
	[11.1; 14.8]	[11.2; 14.2]	[9.5; 12.3]	[13.0; 16.6]	[11.7; 14.0]
**Zeaxanthin** (μg/dl)	2.9 ± 1.4	2.8 ± 1.3	2.7 ± 1.2	3.0 ± 1.5	2.8 ± 1.4
	(2.6)	(2.5)	(2.4)	(2.6)	(2.6)
	[2.5; 3.2]	[2.5 ; 3.2]	[2.4; 3.0]	[2.6; 3.4]	[2.6; 3.1]
**Lutein + zeaxanthin** (μg/dl)	16.5 ± 8.1	15.6 ± 6.6	14.3 ± 6.5^b^	17.9 ± 7.8^b^	16.1 ± 7.4
	(14.6)	(14.1)	(12.8)	(15.7 )	(14.4)
	[14.3; 18.8]	[13.8; 17.5]	[12.5; 16.1]	[15.7; 20.0]	[14.7; 17.5]
**Lutein + zeaxanthin/cholesterol + triglycerides** (μg/mg)	0.06 ± 0.03	0.06 ± 0.03	0.06 ± 0.03	0.06 ± 0.03	0.06 ± 0.03
	(0.05)	(0.06)	(0.05)	(0.05)	(0.05)
	[0.05; 0.06]	[0.06; 0.07]	[0.05; 0.07]	[0.05; 0.07]	[0.06; 0.07]
**Cholesterol** (mg/dl)	186.0 ± 42.9	187.1 ± 42.9	165.4 ± 43.2^b^	207.7 ± 43.1^b^	186.6 ± 48.0
	(178)	(191)	(163.5)	(201.0)	(188)
	[174.3; 195.7]	[172.7; 2016]	[153.6; 177.2]	[196.0; 219.5]	[177.4; 195.7]
**HDL cholesterol** (mg/dl)	52.5 ± 14.1^a^	62.4 ± 12.8^a^	55.6 ± 12.9	59.4 ± 15.5	57.5 ± 14.3
	(51.5)	(65.5)	(54)	(60.0)	(56)
	[48.7; 56.4]	[58.9; 65.9]	[52.1; 59.1]	[55.1; 63.6]	[54.8; 60.2]
**LDL cholesterol** (mg/dl)	110.7 ± 37.2	109.7 ± 43.6	92.8 ± 35.3^b^	127.6 ± 37.7^b^	110.2 ± 40.3
	(107)	(112.5)	(88 )	(121.5)	(108)
	[100.6; 120.9]	[97.8; 121.5]	[83.1; 102.4]	[117.3; 137.9]	[102.5; 117.9]
**Triglycerides** (mg/dl)	105.7 ± 55.3^a^	74.5 ± 23.9^a^	86.3 ± 36.3	94.0 ± 52.7	90.1 ± 45.2
	(87.9)	(70.5)	(76.0 )	(78.5)	(78)
	[90.6; 120.8]	[68.0; 81.1]	[76.4; 96.2]	[79.6; 108.4]	[81.5; 98.8]
***Dietary intake***					
**Lutein (μg/day)**	955.4 ± 1418.1	1190.8 ± 1727.4	903.9 ± 1571.8^b^	1242.3 ± 1579.3^b^	1073.1 ± 1577.4
	(408.8)	(410.5)	(339.2)	(528.8)	(410.5)
	[568.4; 1342.5]	[719.3; 1662.3]	[474.9; 1333.0]	[811.3; 1673.4]	[772.2; 1374.0]
**Zeaxanthin (μg/day)**	78.6 ± 121.1	111.7 ± 155.7	91.5 ± 143.5	98.8 ± 137.2	95.2 ± 139.8
	(31.8)	(48.9)	(33.0)	(46.3)	(38.1)
	[45.6; 111.7]	[69.2; 154.2]	[52.3; 130.7]	[61.3; 136.2]	[68.5; 121.8]
**Lutein + zeaxanthin (μg/day)**	1052.8 ± 1531.6	1283.7 ± 1860.6	995.4 ± 1705.0^b^	1341.1 ± 1693.1^b^	1168.3 ± 1700.0
	(445.9)	(452.5)	(369.0)	(678.6)	(451.0)
	[634.8; 1470.9]	[775.9; 1791.5]	[530.0; 1460.8]	[879.0; 1803.2]	[844.0; 1492.5]
**Lutein + zeaxanthin/1000 Kcal (μg/day)**	47.5 ± 71.2	66.8 ± 91.5	47.7 ± 78.4^b^	66.6 ± 85.4^b^	57.1 ± 82.2
	(21.2)	(26.0)	(18.8)	(31.9)	(23.8)
	[2.5; 323.9]	[3.5; 379.0]	[2.4; 367.5]	[4.3; 335.3]	[2.56; 329.4]
***Macular pigment optical density*** (density units)					
**MPOD (n = 216 eyes)**	0.352 ± 0.150	0.342 ± 0.155	0.370 ± 0.140^b^	0.325 ± 0.158^b^	0.347 ± 0.108
	(0.360)	(0.350)	(0.360)	(0.320)	(0.360)
	[0.323; 0.382]	[0.311; 0.373]	[0.341; 0.398]	[0.293; 0.356]	[0.326; 0.370]
**Body mass index (kg/m**^**2**^**)**	24.9 ± 2.2^a^	21.8 ± 2.4^a^	22.7 ± 2.8^b^	24.0 ± 2.5^b^	23.4 ± 2.7
	(25.0)	(21.5)	(22.6)	(24.1)	(23.0)
	[24.3; 25.4]	[21.2; 22.5]	[22.0; 23.5]	[23.3; 24.7]	[22.8; 23.9]

Serum values are expressed as mean ± standard deviation (each datum is the mean of three replicate injections), (median) and confidence interval [_95%_ CI].

^a^ Significant difference between sexes (p < 0.0001).

^b^ Significant difference between age groups (p < 0.0005 for all variables, except for BMI p = 0.002).

HDL: high-density lipoprotein; LDL: low density lipoprotein; MPOD: macular pigment optical density.

The difference in lutein and zeaxanthin between the two age groups disappeared when their concentrations were expressed in relation to serum lipids (cholesterol + triglycerides), but was maintained when intake was expressed in terms of dietary energy density (lutein plus zeaxanthin/1000 kcal). Regarding lipids in serum (all the subjects had serum cholesterol levels within normal range), the total cholesterol and LDL-cholesterol concentrations showed age-related differences (higher in older *vs* younger subjects), whereas HDL-cholesterol and triglyceride concentrations showed sex-related differences (the former higher in women than in men and the latter vice versa). The mean BMI in the total sample was within normal range (23.4 ± 2.7 kg/m^2^), being higher in men *vs* women and in older *vs* younger subjects (p = 0.002). Finally, there were differences in the MPOD related to age (lower in older *vs* younger individuals, p = 0.038) but not to sex.

The dietary intake of the major sources of lutein and zeaxanthin (fruit, vegetables and eggs) is shown in Table 2. Energy intake was significantly greater in men than in women (p < 0.001), but this was not the case with the intake of food sources of lutein and zeaxanthin, which only show age-related differences (with the exception of egg consumption), as they were consumed in greater amounts in the older group.

**Table 2:** **Dietary intake of the major sources of lutein and zeaxanthin intake (g/day) and energy intake expressed as mean ± standard deviation, (median) and confidence interval [**_**95%**_** CI]**

	**Men**	**Women**	**Aged 20–35 years**	**Aged 45–65 years**	**Total sample (n = 108)**
***Food groups *** **(g/day)**					
**Fruit**	225.4 ± 197.4	223.4 ± 178.6	150.4 ± 119.5^b^	298.4 ± 213.1^b^	224.4 ± 187.3
	(200.0)	(193.9)	(151.9)	(269.2)	(197.5)
	[171.5; 279.3]	[174.6; 272.1]	[117.8; 183.0]	[240.2; 356.6]	[188.7; 260.1]
**Vegetables**	280.8 ± 158.1	269.1 ± 140.1	238.0 ± 128.4^b^	311.9 ± 159.4^b^	275.0 ± 148.8
	(261.2)	(271.4)	(231.0)	(290.7)	(266.3)
	[237.6; 323.9]	[246.6; 303.3]	[202.9; 273.0]	[268.4; 355.4]	[246.6; 303.3]
**Fruit + vegetables**	506.2 ± 278.2	492.5 ± 265.6	388.4 ± 190.2^b^	610.3 ± 294.6^b^	499.3 ± 270.8
	(451.0)	(475.5)	(410.3)	(577.3)	(458.7)
	[430.3; 582.1]	[420.0; 565.0]	[336.4; 440.3]	[529.9; 690.7]	[447.7; 551.0]
**Eggs**	25.8 ± 28.5	24.8 ± 21.8	25.7 ± 24.8	24.9 ± 26.0	25.3 ± 25.3
	(21.3)	(21.3)	(21.3)	(20.7)	(21.3)
	[18.1; 33.6]	[18.8; 30.7]	[18.9; 32.4]	[17.8; 32.0]	[20.5; 30.1]
**Energy intake (Kcal)**	2334 ± 584^a^	1895 ± 402^a^	2096 ± 494	2133 ± 597	2114 ± 546
	(2233)	(1904)	( 2072)	(1994)	(2054)
	[2174; 2493]	[1785; 2004]	[1961; 2231]	[1970; 2296]	[2010; 2218]

^a^Significant difference between sexes (p < 0.001).

^b^Significant difference between age groups (p < 0.001).

### Correlations between diet and serum concentrations of lutein and zeaxanthin and their major food sources

The concentrations of lutein and zeaxanthin in diet, as well as the amounts of fruit and vegetables consumed, correlated significantly with the concentrations of lutein and zeaxanthin in serum in the total sample (Table 3). On comparing the age groups, those correlations were observed in the younger group but not in the older group (except for zeaxanthin and fruit and vegetable consumption). The intake of lutein and zeaxanthin, expressed as concentration per 1000 kcal, correlated significantly with their concentrations in serum in the total sample, a finding that was due to their correlations in the younger subjects. The highest correlation coefficients were for the correlations between fruit intake and lutein and lutein plus zeaxanthin concentrations in serum (ρ = 0.452, p < 0.0001 and ρ = 0.431, p < 0.0001, respectively) in the younger group.

**Table 3:** **Correlations (Spearman’s ρ and (*p *** **value)) between lutein, zeaxanthin and major food sources for dietary intake and serum concentrations**

***Serum***	**Aged 20 – 35 years**			**Aged 45 – 65 years**			**Total sample**		
***Dietary intake***	**Lutein**	**Zeaxanthin**	**Lutein + Zeaxanthin**	**Lutein**	**Zeaxanthin**	**Lutein + Zeaxanthin**	**Lutein**	**Zeaxanthin**	**Lutein + Zeaxanthin**
**Lutein**	0.266 (0.052)	0.250 (0.068)	0.274 (0.045)	0.135 (0.332)	0.200 (0.148)	0.160 (0.247)	0.250 (0.009)	0.228 (0.018)	0.257 (0.007)
**Zeaxanthin**	0.296 (0.030)	0.205 (0.136)	0.324 (0.017)	0.156 (0.258)	0.366 (0.007)	0.238 ( 0.083)	0.221 (0.022)	0.313 (0.001)	0.286 (0.003)
**Lutein + zeaxanthin**	0.313 (0.021)	0.281 (0.040)	0.314 (0.021)	0.145 (0.296)	0.227 (0.098)	0.173 (0.212)	0.256 (0.007)	0.212 (0.027)	0.277 (0.004)
**Lutein + zeaxanthin (density)**	0.312 (0.022)	0.286 (0.036)	0.316 (0.020)	0.072 (0.606)	0.201 (0.145)	0.119 (0.390)	0.211 (0.029)	0.195 (0.043)	0.239 (0.013)
**Fruit**	0.452 (0.001)	0.400 (0.003)	0.431 (0.001)	0.154 (0.266)	0.340 (0.012)	0.219 (0.111)	0.381 (0.000)	0.382 (0.000)	0.382 (0.000)
**Vegetables**	0.243 (0.076)	0.205 (0.138)	0.225 (0.101)	0.102 (0.463)	0.199 (0.149)	0.122 (0.379)	0.215 (0.025)	0.214 (0.026)	0.200 (0.038)
**Fruit and vegetables**	0.447 (0.001)	0.399 (0.003)	0.401 (0.003)	0.125 (0.368)	0.317 (0.020)	0.181 (0.189)	0.355 (0.000)	0.353 (0.000)	0.341 (0.000)
**Eggs**	0.055 (0.693)	−0.001 (0.996)	0.096 (0.492)	−0.293 (0.033)	−0.342 (0.012)	−0.327 (0.017)	−0.122 (0.210)	−0.193 (0.047)	−0.124 (0.203)

Egg consumption, a good source of lutein, is lower than the Spanish mean intake (32 g/day) [18], showed a wide variability and had a significant inverse correlation with the serum lutein and zeaxanthin concentrations in the older group. The degree of correlation loses statistical significance if only those individuals who consumed eggs are assessed.

### Correlations of lutein and zeaxanthin in diet and serum and the major sources of their dietary intake with macular pigment optical density

**Figure 1:**
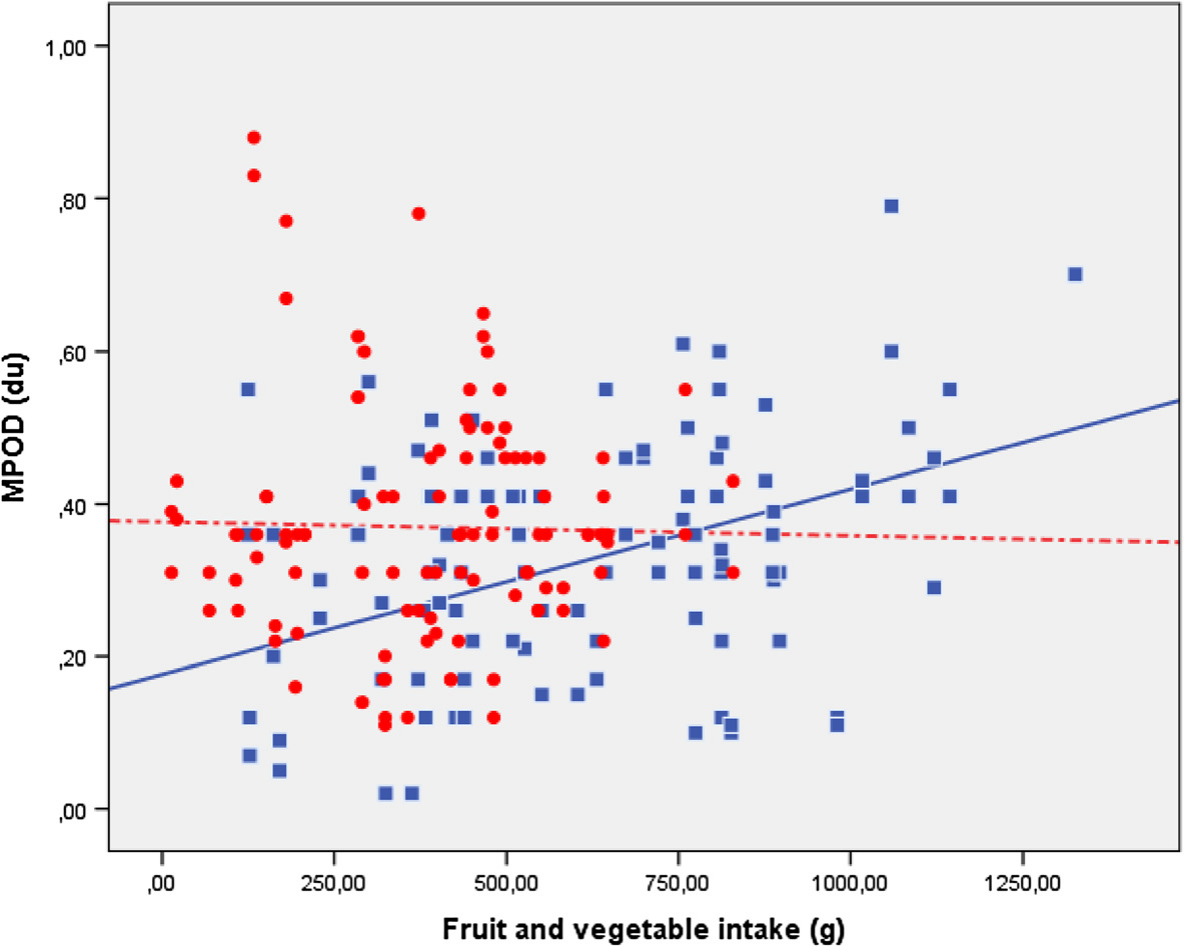
**Correlation between MPOD (du) and fruit and vegetable intake (g) in the younger (circle and dotted line) (r**^**2**^ **= 0.001) and older (square and solid line) (r**^**2**^ **= 0.188) groups.**

As shown in Table 4, the MPOD did not correlate significantly with serum lutein or zeaxanthin in the total sample, but showed significant correlations when they were expressed in relation to cholesterol plus triglycerides (ρ = 0.170, p = 0.012) and to serum LDL cholesterol concentrations (ρ = 0.162, p = 0.017). However, in the older group, in addition to the aforementioned relationships, we observed correlations with lutein, zeaxanthin (ρ = 0.223, p = 0.02) and with lutein plus zeaxanthin when expressed in relation to HDL-cholesterol (ρ = 0.272, p = 0.004) and LDL-cholesterol (ρ = 0.301, p = 0.002). There were no such significant correlations in the younger group.With respect to the relationship between MPOD and the dietary intake of lutein and zeaxanthin, there were no correlations except for that of zeaxanthin in the older subjects and an inverse correlation with the lutein density in the total sample and in the younger group. However, there were significant correlations between fruit and vegetable intake and the MPOD in the older subjects, showing the highest coefficient correlations for the intake of fruit plus vegetables (ρ = 0.350, p = 0.0002), as well as in the total sample (ρ = 0.171, p = 0.012), but no significant correlations were found in the younger group (Figure 1).

**Table 4:** **Statistically significant correlations (Spearman’s ρ and (*p *** **value)) between lutein, zeaxanthin and major food sources for their intake in serum and diet with MPOD (two eyes/subject, n=216)**

	**MPOD**	
	**Aged 45 – 65 years**	**Total sample**
***Serum***		
Lutein	0.204 (0.034)	
Zeaxanthin	0.267 (0.005)	
Lutein + zeaxanthin	0.223 (0.020)	
Lutein + zeaxanthin/cholesterol + triglycerides	0.262 (0.006)	0.170 (0.012)
Lutein/cholesterol + triglycerides	0.258 (0.007)	0.176 (0.009)
Zeaxanthin/cholesterol + triglycerides	0.298 (0.002)	0.152 (0.025)
Lutein/HDL cholesterol	0.239 (0.013)	
Zeaxanthin/HDL cholesterol	0.282 (0.003)	
Lutein + zeaxanthin/HDL-cholesterol	0.272 (0.004)	
Lutein + zeaxanthin/LDL-cholesterol	0.301 (0.002)	0.162 (0.017)
Cholesterol	−0.227 (0.018)	
***Dietary intake***		
Zeaxanthin	0.230 (0.017)	
Lutein (density)		−0.147 (0.030)
Fruit	0.318 (0.001)	0.160 (0.018)
Vegetables	0.255 (0.008)	
Fruit and vegetables	0.350 (0.000)	0.171 (0.012)

MPOD: macular pigment optical density; HDL: high-density lipoprotein; LDL: low density lipoprotein.

The MPOD showed age-specific correlations with lutein plus zeaxanthin expressed in relation to serum lipid concentrations as well as with the fruit and vegetable intake.

### Multivariate regression analysis of dietary and biochemical factors associated with the MPOD

Table 5 corresponds to the regression model used to evaluate the predictive value of age, sex, lutein and zeaxanthin in diet and serum, also expressed in relation to serum lipids, cholesterol (total, HDL- and LDL-), triglycerides and fruit and vegetable intakes on the MPOD value. The table shows only results that do not include zero in the confidence interval. In the total sample, age and serum lutein were the main predictors of MPOD. The R^2^ in the total sample was 11.2% (predictive variables: grams of fruit, age, lutein + zeaxanthin/HDL and serum lutein). However, upon analysis of the sample of older subjects, the main predictor was the concentration of lutein plus zeaxanthin in serum related to the concentration of lipids (cholesterol plus triglycerides) and, to a lesser extent, sex (in this group, the men had higher MPOD than the women). The R^2^ in the older subjects was 29.7% (predictive variables: sex, fruit in grams, vegetables in grams and lutein + zeaxanthin/cholesterol + triglycerides).

**Table 5:** Multivariate regression analysis of biochemical and dietary factors, sex and age data associated with MPOD

	**β (SE)**	***p***	_**95% **_**CI**
***Total sample***			
Constant	0.413 (0.036)	0.000	0.342, 0.485
Age	- 0.094 (0.023)	0.000	- 0.139, −0.048
Lutein (serum)	0.008 (0.003)	0.013	0.002, 0.014
***Older group (45–65 y)***			
Constant	0.246 (0.058)	0.000	0.131, 0.361
Sex	- 0.086 (0.027)	0.002	- 0.139, −0.032
Lutein + zeaxanthin/cholesterol + triglycerides	1.165 (0.491)	0.020	0.191, 2.140

MPOD: macular pigment optical density; SD: standard deviation; CI: confidence interval.

## Discussion

Lutein status is routinely assessed together with zeaxanthin, using dietary or biochemical methods, both of which have advantages and limitations, although serum concentration has been considered the best method for establishing their nutritional status in humans [3]. Different factors linked either to the subject or to diet can influence their concentrations in serum and their bioavailability [3,7,10]. For this report, we selected a sample of apparently healthy subjects with similar serum lipids, body weight and dietary habits (varied diet and no dietary supplements) from two different age groups having the same proportion of men and women. To our knowledge, this is the first study to simultaneously assess dietary and status markers of lutein and zeaxanthin (diet, serum and MPOD) in Spanish subjects taking into account several confounding factors.

Serum lutein and zeaxanthin concentrations are similar to those reported in other groups of Spanish subjects [12] and somewhat lower than [4,10] or similar to [1,19,20] those of comparable age in several European and North American countries. Serum lutein concentrations in the present study, and in practically all those mentioned above, are much lower than the >34 μg/dL (0.6 μmol/L) that seem to be consistently associated with lower risk in epidemiological studies (including lower risk for age-related macular degeneration and cataracts) and higher MPOD [3].

We observed no sex differences in serum concentrations and dietary intake of lutein and zeaxanthin or in MPOD, findings reported in a number of studies [4,20-22], although other authors observed higher intake in women [23]. In contrast, although cholesterol was within normal range, there were differences in serum lipids, and higher triglyceride and lower HDL-cholesterol levels were observed in men. However, serum lutein and zeaxanthin concentrations differed according to the age group, being higher in the older subjects. Their intake, both crude and energy-adjusted, is also greater, as reported elsewhere [24], and this group consumes more fruits and vegetables, as well. These differences disappear when serum concentrations are related to levels of circulating lipids (lutein + zeaxanthin/cholesterol + triglycerides), transporters of these xanthophylls, which are being considered for a better interpretation of the antioxidant/nutritional status [11,25] or are expressed as 1000 kcal intake. The MPOD in the total sample was 0.35 du, similar to that of other studies [21,22,26], and differed according to age [17].

However, the amount of lutein plus zeaxanthin intake (median value) is much lower than that found by our group more than a decade ago in European subjects (n = 400) [27]. In that study, the intake in the Spanish subgroup (n = 80) was 3.25 mg/day (range: 1.8-4.3 mg/d), which contrasts with the 0.45 mg/d of this study, which, in turn, is similar to the mean intake in the Spanish population obtained in national surveys (0.5 mg and 0.1 mg/person/day of lutein and zeaxanthin, respectively) [28]. It is also similar to the levels reported in other populations [10]. If we compare the mean intake usually found in the literature, rather than the median value (preferable since the data usually do not follow a normal distribution), the mean intake of 1.2 mg is similar or slightly below the range described in other studies [6,8,21,24].

The different intake levels found in the literature are largely attributable to the differences among databases for carotenoid composition in foods and the types of dietary questionnaires employed [12]. Regarding the aforementioned European study, the food composition data utilized are very similar to those used in this report [15] since, in both cases, much of the data resulted from HPLC analysis of Spanish foods; however, the European study was based on a semiquantitative food frequency questionnaire (FFQ), whereas in the present study, 3-day food records were employed. FFQ have been reported to overestimate carotenoid intake [23], especially that of lutein and zeaxanthin when comparing these two methods [12]. FFQ were used in those studies in which intake concentrations were higher and 2 or 3-day recalls were employed in others in which the concentrations were comparable. On the other hand, the data from population-based studies in the USA and Spain indicate that levels of lutein and zeaxanthin intake have declined, particularly from dark-green leafy vegetables [29]. Another aspect to be considered regarding lutein intake is food seasonality; however, in the Spanish population, the mean intake of lutein has been reported to be relatively constant throughout the year [28].

Concerning the sex differences in lutein and zeaxanthin intake, the results in the literature are inconsistent, indicating a higher intake among women [22,23], in whom a lower energy intake [23] or no differences [21] are likewise reported. There are also discrepancies in the findings according to age group, with some studies that show no differences [4] and others that do; in the later, as in our study, a higher intake is reported for older individuals [24].

Lutein intake is higher than that of zeaxanthin in all populations [6,20]; in a typical western diet, a ratio of 7:1 (lutein:zeaxanthin) has been reported [8]. This is lower than that found in our study, which is 11:1 in the total sample and 13:1 in the older subjects, who consume more fruit and vegetables (Table 2) than the younger participants (10:1). The mean intake of fruits and vegetables is higher than that recommended by the WHO to decrease risk of chronic diseases (400 g/day).

The degree of correlation between lutein and zeaxanthin concentrations in serum and diet is significant, being higher if determined by dietary intake of fruit and vegetables, rather than by measuring the concentrations of these xanthophylls as provided by the diet (Table 3). The Pearson correlation coefficient between their serum concentrations and dietary intake is >0.2 and is >0.3 when serum concentrations correlate with food consumed, as in other studies [8,21,22,26]. Aside from the amount injested, the degrees of correlation between biological markers, apparently low, are influenced by many other subject-related factors (i.e. sex, age, BMI, eating habits, sample size), as well as metabolic factors [2,8], and in our study, the degrees of correlation were found to vary according to age group, but not sex. Thus, in younger subjects, there is a correlation between serum levels and dietary intake (in terms of both the concentrations of these compounds intake density) whereas, in older subjects correlation was observed only for zeaxanthin, despite the fact that these individuals have higher levels both in their intake and in serum. Therefore, other metabolic factors must influence metabolism of these xanthophylls, such as the uptake of lutein and zeaxanthin from plasma and their transport to the retina (e.g. serum lipid levels, or binding protein in optical tissue with binding capacity for zeaxanthin isomers, but not for lutein) [7]. Correlation in young subjects but not in their elders has been reported by other authors [19]. However, the correlations between serum concentrations and fruit and vegetable intake are significant in both age groups, especially for fruit intake.

The concentration of lutein and zeaxanthin in the retina, assessed by MPOD, was lower in the older subjects, despite the fact that their intake was higher than that of younger individuals (also expressed in relation to 1000 kcal). These higher intakes are reflected in the higher serum concentrations, although the differences disappear when the levels are expressed in relation to serum lipids. Correlations between MPOD and serum lutein and zeaxanthin and dietary intake reached the highest significance level for zeaxanthin and for lutein + zeaxanthin in relation to lipids (cholesterol + triglycerides, LDL-cholesterol and HDL-cholesterol) and when related to diet (lutein density and fruits and vegetables) (Table 4). Lutein and zeaxanthin are transported in LDL and HDL in similar amounts and it is widely accepted that oxidative modifications of LDL and HDL affect lipoprotein metabolism and modified LDL may have an effect on retinal pigment epithelial cells [30].

There are significant correlations in the elderly group, but not in the younger group (except the correlation with the lutein intake expressed as concentration per 1000 kcal, [ρ = −0.214, p = 0.026]). Although, MPOD is often positively associated with dietary and serum lutein and zeaxanthin concentrations [26], conflicting results have been reported on the influence of sex [22], age [8], BMI [2,31], and other postprandial or environmental factors (e.g. smoking) [22]. The highest correlations between MPOD and lutein and zeaxanthin in serum and dietary intake correspond to fruit and vegetables (ρ = 0.35) and to lutein + zeaxanthin/HDL-cholesterol (ρ = 0.301); the latter is consistent with the identification of HDL as the specific transporter of lutein and zeaxanthin to retina in chicks [32]. The strong correlation between MPOD and fruit and vegetable intake, also reported elsewhere [26], indicates that not only the amounts of lutein and zeaxanthin are important for ocular tissue; there are other micronutrients and bioactive compounds in these foods that are also beneficial (e.g. fiber and polyunsaturated fatty acid intake are also directly related to MPOD) [33]. On the other hand, few studies have assessed lutein and zeaxanthin status simultaneously using the three possible markers (diet, serum and MPOD) and these studies are not very homogeneous in terms of factors that can exert an influence such as sex, age, serum lipids and BMI, among others [21,22,26]. Nevertheless, although consistent associations among these three markers have not always been found, most studies point to the protective role of a diet rich in fruits and vegetables. However, in multivariate regression analysis, performed to assess the predictive value of lutein and zeaxanthin concentrations in serum and dietary intake, sex, age and the concentrations of different serum lipids for MPOD, only the serum lutein concentration and lutein + zeaxanthin in relation to cholesterol + triglycerides, but not fruit and vegetable intake, correlate with MPOD. Moreover, age is an important predictor in the total sample, as is sex in the older group, although we observed no sex differences in the MPOD values in this study, a fact that could be explained if the availability of lutein and zeaxanthin to the retina could be influenced by physiological sex differences [22] (i.e. if binding protein in optical tissue specific for zeaxanthin responds differently in men and women [7]).

## Conclusions

In conclusion, lutein and zeaxanthin concentrations in serum and dietary intake of these xanthophylls and of their major food sources are higher in older *vs* younger subjects; however, MPOD is lower. In younger individuals, MPOD is influenced by serum lutein but, in older individuals, the presence of lutein plus zeaxanthin in relation to circulating lipids is a determining factor. Thus, when establishing normal/reference ranges for serum lutein and zeaxanthin, age ranges should be taken into account and the levels of these xanthophylls should be expressed in relation to the lipid concentration, at least in subjects over 45 years of age, as this could influence MPOD.

## Abbreviations

MP: Macular pigment; MPOD: Macular pigment optical density; BMI: Body mass index; HDL: High density lipoprotein; LDL: Low density lipoprotein.

## Competing interests

The authors declare that they have no competing interests.

## Authors’ contributions

BOA, CCV and BBdM conceived and designed the study; RES, CCV and BBdM acquired and evaluated the dietary data; RES and BOA analyzed the carotenoids in serum and visual data; BOA and BBdM performed the statistical analysis and wrote the paper. All authors have read and approved the final manuscript.
